# Divergent Roles of RPA Homologs of the Model Archaeon *Halobacterium salinarum* in Survival of DNA Damage

**DOI:** 10.3390/genes9040223

**Published:** 2018-04-20

**Authors:** Jessica J. Evans, Patrick E. Gygli, Julienne McCaskill, Linda C. DeVeaux

**Affiliations:** 1 South Dakota School of Mines and Technology, Biomedical Engineering Program, Rapid City, SD 57701, USA; evanjess.je@gmail.com; 2 Idaho State University Department of Biological Sciences, Pocatello, ID 83209, USA; gyglpatr@isu.edu (P.E.G.); mccajuli@gmail.com (J.M.); 3 New Mexico Institute of Mining and Technology, Department of Biology, Socorro, NM 87801, USA

**Keywords:** haloarchaea, DNA damage, single-stranded DNA binding protein, deletion, ionizing radiation, UV-C, mitomycin C, replication protein A

## Abstract

The haloarchaea are unusual in possessing genes for multiple homologs to the ubiquitous single-stranded DNA binding protein (SSB or replication protein A, RPA) found in all three domains of life. *Halobacterium salinarum* contains five homologs: two are eukaryotic in organization, two are prokaryotic and are encoded on the minichromosomes, and one is uniquely euryarchaeal. Radiation-resistant mutants previously isolated show upregulation of one of the eukaryotic-type RPA genes. Here, we have created deletions in the five RPA operons. These deletion mutants were exposed to DNA-damaging conditions: ionizing radiation, UV radiation, and mitomycin C. Deletion of the euryarchaeal homolog, although not lethal as in *Haloferax volcanii*, causes severe sensitivity to all of these agents. Deletion of the other RPA/SSB homologs imparts a variable sensitivity to these DNA-damaging agents, suggesting that the different RPA homologs have specialized roles depending on the type of genomic insult encountered.

## 1. Introduction

The single-stranded DNA binding proteins (SSBs) and replication protein A (RPA) in archaea and eukaryotes) are ubiquitous elements of replication forks across all three domains of life. In addition to their role in coating and protecting lagging strand DNA, they are necessary for protection of single-stranded regions generated during other processes, including restarting stalled replication forks, homologous recombination, and DNA repair [1,2,3,4]. During these processes, SSB/RPA proteins recruit and interact with a variety of other cellular proteins, and their interactions change depending on the state of the DNA [1,4,5,6]. Through these interactions, SSB/RPAs orchestrate the process being carried out [1,2,4,5,6]. SSBs/RPAs are characterized by containing one or more ssDNA binding domains, oligonucleotide oligosaccharide-binding (OB) fold domains, and are thought to have originated from duplication of an ancestral SSB gene [7,8]. Typical bacterial SSBs are encoded by a single predominant essential gene and function as homotetramers [4,9,10]. Eukaryotic RPAs are heterotrimeric and encoded by genes *RPA1*, *RPA2*, and *RPA3* [7,11]. The Archaea are more diverse in their repertoire. Like bacteria, the Crenarchaea typically contain a single gene with a single OB-fold; however, whether the functional protein is a monomer or multimer is unclear, as the OB-fold is more eukaryotic than prokaryotic [8,12]. The euryarchaea, on the other hand, often contain a unique RPA found only among the euryarchaea, and may possess multiple OB-fold-containing proteins, the most common of which contains two OB-folds and a zinc–finger domain [8,13]. The Haloarchaea, for example, typically contain genes for several types of proteins [8]. A bacterial or crenarchaeal protein with one OB-fold is encoded on the mini-chromosomes. At least one operon encoding proteins with homology to RPA1 and RPA2, as well as a third gene encoding a protein with no homology to RPA3, is found on the main chromosome [8,12]. Finally, the uniquely euryarchaeal RPA contains three to four OB-folds in a single open reading frame [8,12].

The extremely radiation- and UV-resistant model halophile *Halobacterium salinarum* provides a unique system for the study of the individual roles of multiple SSB/RPA proteins. In radiation-resistant mutants of *Hbt. salinarum*, one of the two eukaryotic-like operons present on the main chromosome was constitutively upregulated [14,15]. The three genes, *rfa3*, *rfa8*, and *ral*, were shown to be co-transcribed, and recent evidence has suggested that at least the Rfa3 and Rfa8 proteins interact in the cell [16]. Additional expression of this operon on a plasmid conferred increased radiation resistance, indicating that increased levels of these proteins are sufficient to increase resistance [16].

In this study, we have systematically deleted each of the genes with homology to SSB/RPA in *Hbt. salinarum*, except for the first gene (*rfa2*) in one of two operons encoding eukaryotic-type RPA proteins. The effect of these mutations on growth, as well as the phenotype of these mutants after exposure to DNA-damaging agents, has been tested. Interestingly, despite the high degree of homology between *Haloferax volcanii* and *Hbt. salinarum* regarding these genes, we were unable to delete *rfa2*, whereas the homolog was not essential in *Hfx. volcanii* [17]. The RPA/SSB proteins of the two halophiles and their shared features are shown in Table 1 [17]. However, deletion of the *Hfx. volcanii* homolog conferred DNA damage sensitivity. In contrast, we show that deletion of *rfa1* in *Hbt. salinarum* confers an extreme DNA damage sensitivity, but the gene is not essential, whereas the homolog was shown to be essential in *Hfx. volcanii* [17]. These results suggest that despite the high degree of conservation among the haloarchaea regarding the group of single-stranded DNA binding proteins, there has been divergence of roles between these two related species regarding specific involvement in DNA repair pathways and replication.

## 2. Materials and Methods

### 2.1. Strains, Media, and Growth Conditions

*Halobacterium salinarum* ssp. NRC-1 and derivative mutant strains (Table 2) were grown in CM+ medium or minimal Grey and Fitt medium lacking uracil (GF-U) as appropriate [19,20]. All chemicals were purchased from ThermoFisher Scientific (Hampton, NH, USA) unless otherwise stated. Mevinolin and 5-fluoroorotic acid (5-FOA) were added to CM+ where appropriate for selection. *Escherichia coli* strain DH5α (Invitrogen, Carlsbad, CA, USA) was used for plasmid construction and was grown in LB medium containing ampicillin at a concentration of 100 µg/mL where appropriate [21].

### 2.2. Transformation

*Hbt. salinarum* was transformed by an adaptation of the PEG method [20,22]: Late log-phase cells were harvested by centrifugation, and cell pellets were washed with spheroplasting solution (SPS; 2 M NaCl, 25 mM KCl, 50 mM Tris pH 8.8, 15% sucrose) then resuspended gently in fresh SPS. The S-layer was removed by addition of 0.5 M EDTA in SPS. Approximately 1 µg plasmid DNA in 2 M NaCl was added to the cells and incubated for 5 min. SPS containing 60% PEG-600 was added to the caps of each tube and vigorously mixed. Samples were incubated for 15 min, then recovery medium (CM+ containing 15% sucrose) was added, and samples were centrifuged. The supernatant was decanted, and samples were washed twice in recovery medium. Pellets were then resuspended in recovery medium and shaken at 37 °C for 1 to 3 days to allow for recovery of the cells. Cells were then centrifuged, resuspended in basal salts solution (BSS, 4.3 M NaCl, 81 mM MgSO_4_, 14 mM KCl, 5 mM Na_3_C_6_H_5_O_7_) or CM+, and plated on the appropriate growth medium. Plates were incubated at 42 °C for 7–21 days until colonies formed.

*E. coli* DH5α was made competent using calcium chloride treatment and transformed using established methods [23].

### 2.3. Plasmids and Strain Construction

Plasmid pMPK410 [24] was used to make uracil auxotrophic strains of *Hbt. salinarum* ssp. NRC-1 and derivative strain LH5 [14]. Plasmid DNA was transformed as described, and transformants were plated on CM+ containing mevinolin for selection of the integrated plasmid. Transformant colonies were streaked twice on GF-U, then grown to stationary phase in liquid CM+. Diluted cultures were spread on CM+ containing 5-FOA. Plates were incubated at 42 °C for 7–10 days for selection of de-integration of the plasmid and loss of the *ura3* gene. Colonies were then purified once on CM+ with 5-FOA and once on CM+, and uracil auxotrophy was confirmed by plating on GF-U. Deletion of the *ura3* gene was confirmed via PCR.

Plasmids for RPA gene deletion were constructed to generate in-frame deletions of each gene. Briefly, primers pairs were designed to amplify ~500 bp of upstream and ~500 bp of downstream sequence for homology for integration (Supplementary Table S1). One primer in each pair contained one engineered *Eco*RI site for cloning into the suicide vector. The primers bridging the deletion were designed with overlapping sequences. The upstream and downstream region amplicons were combined to create deletion fragments by overlap extension PCR. The resulting deletion fragments were confirmed by gel electrophoresis, then digested in PCR buffer with *Eco*RI (Fermentas, Waltham, MA, USA) followed by purification using the MiniElute PCR cleanup kit (Qiagen, Germantown, MD, USA). Digested deletion fragments were ligated to purified *Eco*RI-digested and dephosphorylated pMPK408 [24]; the ligation reaction was then transformed into DH5α. Recombinant plasmids were isolated from transformants using an alkaline lysis miniprep kit (Stratagene, San Diego, CA, USA), analyzed by restriction digestion using *Eco*RI, and verified through PCR.

Deletions of RPA genes were generated in a similar manner as uracil auxotrophs with the following changes. Transformants were plated on GF-U for selection of the integrated plasmid and grown at 42 °C for 7–10 days. Transformants were purified twice on GF-U, then grown to stationary phase in two separate cultures in either CM+ or GF-U. Presence of the integrated plasmid was confirmed by PCR amplification using DNA from cultures grown in GF-U to detect both the deletion allele and the wild-type allele using the appropriate upstream (UF) and downstream (DR) primers (Supplementary Table S1). The cultures grown in CM+ were plated on CM+ containing 5-FOA to select for loss of the integrated plasmid. Plates were incubated at 42 °C for 7–10 days. Colonies were then purified once on CM+ containing 5-FOA, then once on CM+. Purified colonies were grown in liquid CM+, and cells were harvested by centrifugation. Pellets were lysed in deionized water, and this lysate was used as DNA template for PCR amplification. Deletion of each RPA was confirmed by amplification with the appropriate UF and DR primers (Supplementary Table S1) followed by gel electrophoresis to screen each culture for either the deletion or wild-type allele.

### 2.4. DNA Damage Treatments

#### 2.4.1. Ionizing Radiation

All irradiations were performed at the Idaho Accelerator Center (Idaho State University, Pocatello, ID, USA) using a pulsed S-band medical grade linear accelerator (LINAC) delivering 23 MeV electrons at 60 Hz with a pulse-width of 2 µs as described previously [25]. A 200 μL cell suspension in CM+ medium (approximately 1 × 10^7^ cells) was irradiated at room temperature in polypropylene PCR tubes (Molecular Bioproducts, San Diego, CA, USA). After irradiation, cultures were 10-fold serially diluted to 10^−5^ in liquid CM+ and 10 μL of each dilution was spotted on a CM+ plate and allowed to dry. Plates were incubated at 42 °C for 7 days then analyzed for survival.

#### 2.4.2. Ultraviolet Irradiation

Early log-phase cultures were serially diluted to 10^−5^ in liquid CM+, and 10 μL of each 10-fold dilution was spotted on a CM+ plate and allowed to dry. Plates were exposed to 254 nm UV-C at a power of 1.5 W/m^2^ for various times as indicated in each experiment. Treatment was performed in the dark to remove the effects of photoreactivation, and plates were incubated at 42 °C in the dark for 7 days, then analyzed for survival. For each strain, surviving fractions from triplicate experiments were averaged and plotted in Origin 2018 (OriginLab, Northampton, MA, USA) as described previously, with error bars representing the standard error of the mean [14].

#### 2.4.3. Mitomycin C Treatment

Mitomycin C was added at a final concentration of 0.75 µg/mL to exponential phase broth cultures and incubated at 42 °C with shaking for 20 min. After exposure, cultures were pelleted and resuspended in fresh medium for recovery, then diluted and plated on solid CM+ as appropriate.

### 2.5. Reverse Transcription—Polymerase Chain Reaction and Transcript Analysis

cDNA was made using a microarray labeling and hybridization protocol developed previously [26]. Briefly, in a nuclease-free PCR tube, 10 µL of RNA and 1 µL of random hexamers were mixed thoroughly and the total volume was brought up to 16 µL. This reaction was incubated at 65 °C in a thermocycler for 10 min. Then, 9.8 µL of RT master mix (6 µL of 5X 1st strand buffer; 3 µL of 0.1 M dithiothreitol (DTT); 0.6 µL of deoxyribonucleotide triphosphate (dNTP) mix (25 mM dATP, dTTP, dGTP, and 10 mM dCTP); 0.2 µL of RNase out ribonuclease inhibitor, all chilled on ice for 2 min) was added to the previous reaction. Then, 2 µL of Superscript III Reverse Transcriptase (Invitrogen) was added, mixed by pipetting, and incubated for 10 min at room temperature. The reaction was then incubated at 42 °C in a thermal cycler for 110 min with the lid temperature set at 43 °C. The reaction was stopped by adding 1.5 µL 1 M NaOH and placed in a thermocycler at 65 °C for 10 min. The reaction was stopped with 1.5 µL 1 M HCl and quickly diluted with 150 µL neutralize tagment (NT) buffer from a NucleoSpin Extract II kit (Macherey-Nagel, Düren, Germany). The reactions were purified with the same clean up kit and eluted with nuclease free water.

Specific transcripts were amplified by RT-PCR using transcript primers (Supplementary Table S2). DNA, RNA, and cDNA were used as template. For the reactions with the primers amplifying the *rfa2/rfa7* junction, the following parameters were used: 1 cycle of 98 °C for 30 s; 29 cycles of 98 °C for 10 s, 66 °C for 10 s, and 72 °C for 15 s; and 1 cycle of 72 °C for 5 min. For the reactions with the primers amplifying the *rfa7/yhcR* junction: 1 cycle of 98 °C for 30 s; 29 cycles of 98 °C for 10 s, 63 °C for 10 s, and 72 °C for 15 s; and 1 cycle of 72 °C for 5 min.

### 2.6. Quantitative PCR

RNA was isolated from 5 mL of culture with an Invitrogen PureLink™ RNA Mini Kit. To ensure that no residual DNA was present, the Invitrogen™ Ambion™ TURBO DNA-*free* kit was also used before conversion to cDNA. Concentrations of each cDNA sample were determined using a ThermoFisher Scientific NanoDrop 2000, and 2 µg of template was used in the Applied Biosystems (Foster City, CA, USA) High Capacity RNA-to-cDNA kit.

All qPCR reactions were performed in with three biological and three technical replicates on a PikoReal Real Time PCR System (ThermoFisher Scientific) with TaqMan^®^ Gene Expression primers and probes (Supplementary Table S3) under the following conditions: 95 °C for 10 min; (95 °C for 1 min, 60 °C for 30 s) for 35 cycles; and 72 °C for 7 min [26]. Analysis was completed using PikoReal Software 2.2 (ThermoFisher Scientific).

Standard curves were obtained using serially diluted gene targets of known concentrations that were amplified via PCR (Supplementary Table S1). Triplicate samples of each dilution were used as template in qPCR and the recorded Cq was graphed versus DNA concentration. Standards for the gene target were included with all experimental sample plates to ensure consistent readings.

### 2.7. Statistical Analysis

For colony size analysis and DNA damage experiments, statistical significance was determined by analysis of variance (ANOVA) with post-hoc Tukey Honestly Significant Difference (HSD) test. Details are noted in the figure captions for each experiment.

For quantitative PCR analysis, statistical significance was determined using R version 3.4.2. [27]; a two-factor ANOVA followed by the post-hoc Tukey HSD test was performed for each gene, and a one-way ANOVA followed by the post-hoc Tukey Honest Significance Difference test was performed for each gene’s treatment and to determine significance between absolute levels of each gene during normal growth.

## 3. Results

**Figure 1:**
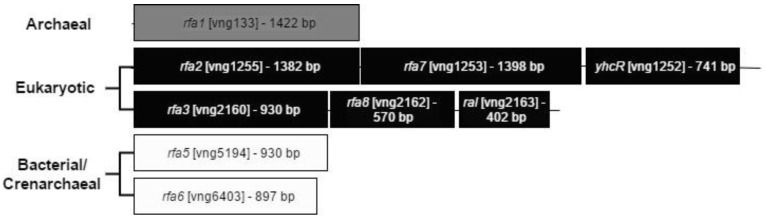
Operon structure and relatedness of the RPA homologues in *Hbt. salinarum.* The Archaeal homolog Rfa1 contains three OB-folds, and is unique to the euryarchaeal lineage [8] The two eukaryotic homologs are each found in three-gene operons, with the first and second genes showing homology to eukaryotic RPA1 and RPA2, respectively. The three genes of the *rfa3* operon (*rfa3*, *rfa8*, and *ral*) have been shown to be co-transcribed [14]. Similar evidence for the *rfa2* operon is presented in this report.

To determine the roles of each of the SSB/RPA homologs present in the genome of *Hbt. salinarum*, an in-frame deletion allele of each gene was created in a *Halobacterium* suicide vector containing a selectable *ura3* gene, and recombinant integrants were selected in an otherwise wild-type Δ*ura3* background (LH101). From these integrants, 5-FOA^r^ excisants were selected, where the integrated plasmid had been lost through a second recombination event (the “pop-in, pop-out” method; [24]). Except for Δ*rfa2*, two excisants—one containing the deletion allele and one containing the restored wild-type allele—were purified for each gene. Each was verified through PCR using the forward primer from the upstream region and the reverse primer from the downstream region. The individual genes deleted were: of the two eukaryotic-type RPAs, each gene within the *rfa3* operon (*rfa3*, *rfa8*, and *ral*) and the second and third genes in the putative *rfa2* operon (*rfa7* and *yhcR*); the uniquely euryarchaeal *rfa1*; and each of the duplicate SSB-type genes on the mini-chromosomes, *rfa5* and *rfa6*. (Figure 1). In addition, qPCR was performed on LH101 (no deletion), LH128 (Δ*yhcR*), LH134 (Δ*rfa3*), LH136 (Δ*rfa8*), LH140 (Δ*rfa1*), and LH142 (Δ*rfa6*) to verify lack of expression, and all levels were below detection (data not shown).

### 3.1. Effect of Replication Protein A Deletions on Growth

#### *rfa1*, *rfa3* and *rfa8* Deletions Affect Growth

**Figure 2:**
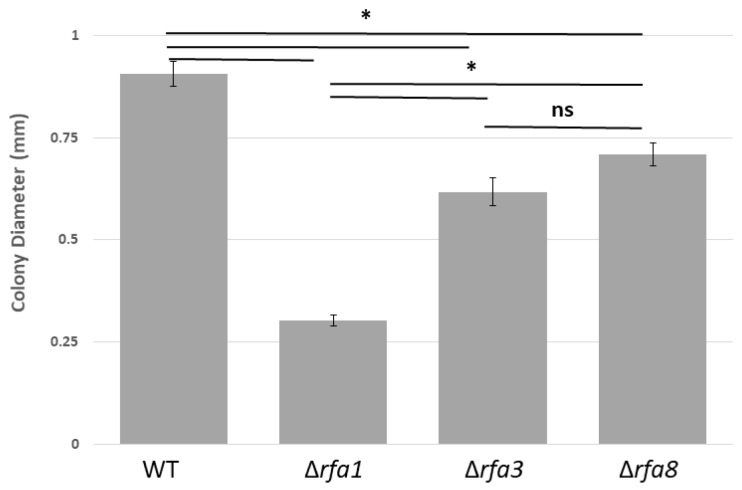
Colony size comparison between LH101 (wild-type: WT) and LH140 (Δ*rfa1*), LH134 (Δ*rfa3*), and LH136 (Δ*rfa8*). Plates were incubated for 7 days. Colony diameter was measured using ImageJ (Version 1.8.0_112, National Institutes of Health, Bethesda, MD, USA) [28]. * *p* < 0.05, analysis of variance (ANOVA) with Tukey’s Honest Significant Difference (HSD), *n* = 22–40 colonies total over 2 biological replicates. Error bars represent the standard error of the mean (SEM). ns = not significant.

The strain carrying a deletion of *rfa3* (LH134), but not *rfa8* (LH136) or *ral* (LH138) within the *rfa3* operon, as well as the strain carrying Δ*rfa1* (LH140), showed reduced colony size in comparison to their isogenic counterparts. This discrepancy in growth can be seen in Figure 2, where the diameter of colonies of strains carrying each of the three deletion alleles are compared to wild-type. Strains carrying Δ*ral* (LH138), Δ*rfa7* (LH166), Δ*yhcR (*LH128), Δ*rfa5* (LH110), or Δ*rfa6* (LH142) also formed normal-sized colonies, and are not shown. The growth defect was more pronounced in LH140 (Δ*rfa1*) than in LH134 (Δ*rfa3*).

### 3.2. Deletion of rfa2, rfa7, and yhcR

#### 3.2.1. Co-Transcription of the *rfa2*, *rfa7*, and *yhcR* Genes

**Figure 3:**
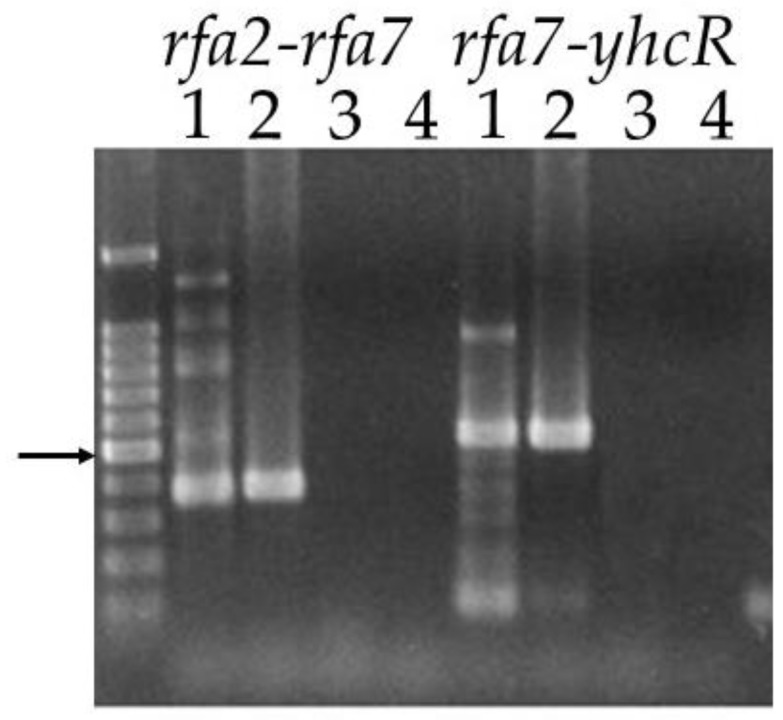
Co-transcription of *rfa2*, *rfa7* and *yhcR*. Primers bracketing the intergenic regions *rfa2-rfa7* and *rfa7-yhcR* were used in reactions with different templates. Lane 1: cDNA; Lane 2: genomic DNA; Lane 3: total RNA; Lane 4: no template. Size marker is 1 kb PLUS DNA ladder (Gold Biotechnology, St. Louis, MO, USA). Arrow indicates 500 bp.

By comparison to the *rfa3* operon, and from sequence analysis, *rfa2*, *rfa7*, and *yhcR* appear to also potentially form an operon. Prior to attempting to create deletion strains, we performed transcript analysis using primers that would allow for amplification of the two intergenic regions, *rfa2-rfa7* (383 bp) and *rfa7-yhcR* (541 bp), from cDNA made from total RNA. The predicted size amplicon was present for both regions when either cDNA or genomic DNA, but not total RNA, was used as template, demonstrating the existence of a transcript containing the intergenic region, and thus confirming co-transcription of the three genes under normal growing conditions (Figure 3).

#### 3.2.2. *rfa2* May Be Essential

Although in *Hfx. volcanii*, strains carrying a deletion of the *rfa2* homolog were obtained [17], we were unable to isolate strains in a wild-type background that carried this deletion in *Hbt. salinarum*. Over 1000 FOA^r^ excisants were analyzed, including those with extremely compromised growth, and all contained the wild-type allele. Strains carrying deletions in either *rfa7* or *yhcR* were readily isolated. Significantly, the Δ*rfa2* deletion allele was recovered in LH102, which is the Δ*ura3* derivative of a previously described radiation-resistant mutant, LH5 [14]. LH5 has been shown to have increased expression of the *rfa3* operon; this increased expression appeared to compensate for the defect imposed by the deletion of *rfa2*. This construct was verified as the others, using the forward primer of the upstream region and the reverse primer from the downstream region and verifying that the deletion allele (1053 bp) instead of the wild-type allele (2581 bp) was amplified (Supplementary Figure S1). However, qPCR analysis of LH154 showed reduction, but not absence, of *rfa2* transcript in this strain (Table 3). Given the fluid nature of the *Hbt. salinarum* genome and its many active insertion elements [29], it is possible that a region amplifiable only by the internal, but not external, primers was rearranged to another transcriptional unit within the chromosome.

### 3.3. Effect of Deletions on Survival to Ionizing Radiation, Ultraviolet C, and Mitomycin C

#### 3.3.1. Δ*rfa3*, Δ*rfa8*, and Δ*rfa1* Confer Ionizing Radiation Sensitivity

Because upregulation of the *rfa3* operon has been shown to confer increased ionizing radiation resistance, but not increased resistance to UV [30], strains containing deletions were tested for survival after exposure to 23 MeV electrons from a LINAC, and UV-C, treatment.

**Figure 4:**
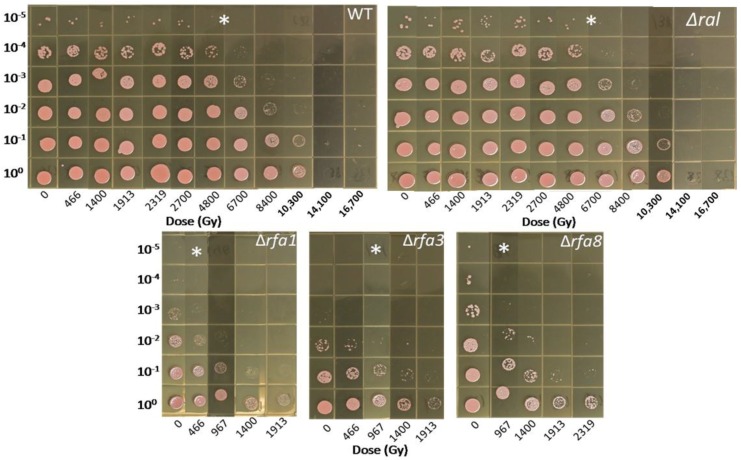
Survival of *Hbt. salinarum* strains to Ionizing radiation exposure. LH101 (WT), LH138 (Δ*ral*), LH140 (Δ*rfa1*), LH134 (Δ*rfa3*), and LH136 (Δ*rfa8*) were exposed to increasing doses of electron beam radiation. * indicates dose at which each strain reaches the approximate D_10_. LH110 (Δ*rfa5*), LH142 (Δ*rfa6*), and LH128 (Δ*yhcR*) showed wild-type survival and are not shown.

Deletion strains were tested in exponential phase against the treatments as described. Based on the involvement of *rfa3* upregulation in ionizing radiation resistance, it was expected that deletion of the genes would affect survival. As seen in Figure 4, strains harboring deletions in either *rfa3* or *rfa8*, but not *ral*, had reduced survival to e-beam exposure compared to wild-type (representative wild-type shown), with comparable 10-fold reductions (D_10_) in survival at approximately 1000 Gy versus approximately 7000 Gy in the wild-type. The strain carrying Δ*rfa1* had a more severe survival defect, with a D_10_ of approximately 500 Gy. All other deletion strains tested (Δ*rfa5*, Δ*rfa6*, Δ*yhcR*) had survival comparable to the wild-type (data not shown). LH166 (Δ*rfa7*) was not available for testing.

#### 3.3.2. Ultraviolet C Survival is Similar to Ionizing Radiation

**Figure 5:**
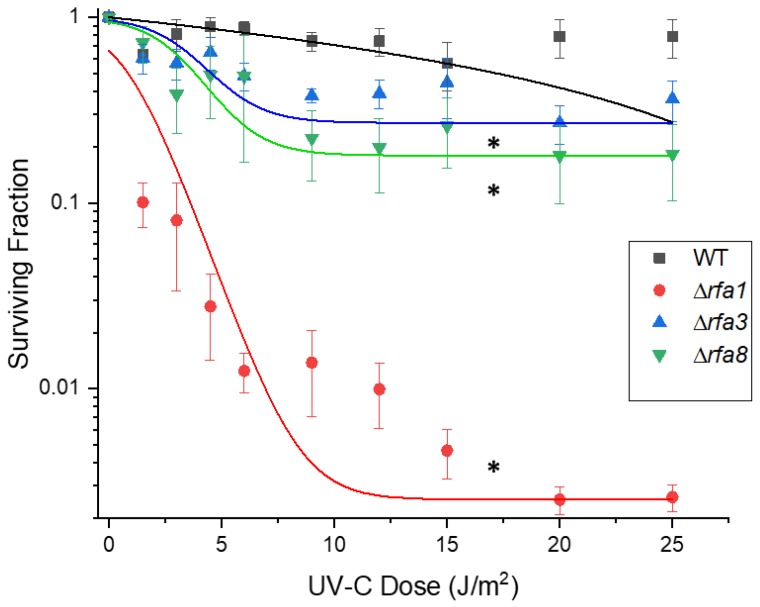
Survival of *Hbt. salinarum* deletion strains to UV-C damage. LH101 (WT), LH140 (Δ*rfa1*), LH134 (Δ*rfa3*), and LH136 (Δ*rfa8*). * *p* < 0.05 compared to LH101; ANOVA with Tukey’s HSD, *n* = 3. Error bars represent +/− standard error of the mean. Mutant strain survival curves were fitted to the Boltzmann function in Origin 2018 (OriginLab, Northampton, MA, USA).

Each strain, as well as LH166 (Δ*rfa7*), were also tested against UV-C; survival showed a similar pattern to IR. The *rfa3* and *rfa8* deletion strains had moderately reduced survival, with 10% survival at approximately 25 J/m^2^ (Figure 5). The *rfa1* deletion strain (LH140) had extremely reduced survival, with 10% survival at approximately 2 J/m^2^. All other deletion strains (Δ*ral*, Δ*rfa7*, Δ*yhcR*, Δ*rfa5*, and Δ*rfa6*) had wild-type levels of survival (Supplementary Figure S2).

#### 3.3.3. Δ*rfa5*, Δ*rfa6,* Δ*rfa7*, and Δ*yhcR* Strains Are Also Sensitive to Mitomycin C

**Figure 6:**
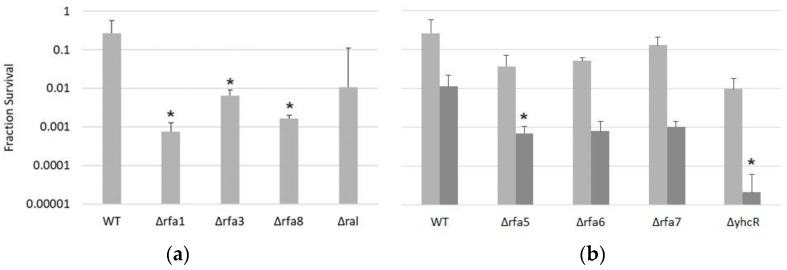
Survival of *Hbt. salinarum* deletion strains to mitomycin C treatment. (**a**) Survival after 30 min exposure for LH101 (WT), LH140 (Δ*rfa1*), LH134 (Δ*rfa3*), LH136 *(*Δ*rfa8*), and LH138 (Δ*ral*). (**b**) Survival after 30 (light grey) and 120 min (dark grey) exposure for LH101 (WT), LH110 (Δ*rfa5*), LH142 (Δ*rfa6*), LH166 (Δ*rfa7*), and LH128 (Δ*yhcR*). * *p* < 0.05 compared to LH101, ANOVA with Tukey’s HSD, *n* = 3. Error bars represent +/− standard error of the mean.

Given the overlapping repair pathways for IR and UV damage, it was not unexpected that certain deletions would show similar phenotypes when challenged with those. To test an additional type of damage, DNA crosslinking, all deletion strains were treated with mitomycin C and survival at various times post-treatment was measured. Similarly to the IR and UV treatments, the Δ*rfa1,* Δ*rfa3*, and Δ*rfa8* strains were the most affected by this treatment and did not survive past 30 min exposure, while Δ*ral* showed no significantly increased sensitivity (Figure 6a). Interestingly, although LH110 (Δ*rfa5*) and LH42 (Δ*rfa6*) showed no difference in growth rate, colony size, IR-resistance, or UV resistance compared to the wild-type, mitomycin C treatment induced a ~15-fold decrease in survival in both deletion strains compared to the wild-type (Figure 6b). Even more drastic was the ~100-fold decrease in survival at 120 min of LH128 (Δ*yhcR*), but not LH166 (Δ*rfa7*).

### 3.4. Expression Levels of Replication Protein A Genes

#### 3.4.1. Replication Protein A Genes are Expressed Under Normal Growing Conditions

The DNA damage survival results suggest that the different RPA genes in *Hbt. salinarum* are involved in several aspects of DNA metabolism and repair. One significant question is whether all are expressed under normal growing conditions, or whether the redundancy of homologs includes non-transcribed genes. The *rfa3* operon has been shown to be upregulated in response to UV [31,32], but absolute expression of the RPA genes has not yet been established. We sought to establish the basal level of transcription of the genes/operons in wild-type *Hbt. salinarum* as well as regulation under DNA-damaging conditions. Levels of the third genes in the two multigene operons, *ral* and *yhcR*, were not measured. Because of the high level of identity between *rfa5* and *rfa6*, these were measured together using one primer pair.

Absolute expression was determined for each gene target during early log-phase normal growth. These values are presented in Table 4, with the housekeeping gene *eef2* as control. *rfa5*/6 and *rfa2* were expressed at the lowest, but still detectable, levels. The *rfa3* operon (*rfa3* and *rfa8*) was the most highly expressed, with levels comparable to *eef2*.

Interestingly, *rfa7* levels were 5-fold higher than *rfa2* levels despite the co-transcription. This suggests a potential second promoter for *rfa7* and a function distinct from *rfa2*.

#### 3.4.2. DNA-Damaging Treatment Results in Differential Expression

It has previously been reported from microarray analysis that *rfa3* operon expression increased following IR and UV treatment [14,22,32]. We measured absolute levels of mRNA, as above, three hours after separate treatment with UV-C and mitomycin C. The *rfa3* operon genes were modestly, but not statistically significantly, upregulated following UV treatment, and similarly following mitomycin C treatment (Table 5). Much more striking was the seven-fold upregulation of *rfa2*, but not *rfa7*, following both UV and mitomycin C treatment. A modest upregulation of *rfa5*/6 following mitomycin C treatment was only slightly below the 95% confidence level, and may indicate a response in transcription to the crosslinking damage, suggesting a role for these homologs in that specific repair pathway.

The upregulation of *rfa5* and *rfa6* following treatment with mitomycin C is in line with the sensitivity of the corresponding deletion strains to this damaging agent. This suggests a specific role in the repair pathway for the damage inflicted by mitomycin C, and provides a reason for the retention of these genes in the genome. The upregulation of *rfa2*, but not *rfa7*, following both UV-C and mitomycin C treatment supports the independent roles of these co-transcribed genes in the damage response.

## 4. Discussion

In this report, we have successfully utilized an established method to individually replace wild-type genes encoding RPA homologs of the haloarchaeon *Hbt. salinarum* with engineered deletions. Although each deletion was constructed to remove only the coding region of each gene, it is possible that polar effects were exerted on remaining genes within the operon. However, we consider this unlikely, as deletion of each individual gene had a unique phenotype. For example, in the *rfa3* operon, deletion of *rfa3* and *rfa8* distinctly affected radiation survival, whereas deletion of *ral* did not. Similarly, in the *rfa2* operon, *rfa7* and *yhcR* deletions affected mitomycin C survival, and a deletion of *rfa2* could not be recovered. Given the ease of generating these deletions, it is unlikely that second-site suppressors have masked the essentiality of these genes. Polarity effects as well as essentiality questions could be addressed by providing the deleted gene in trans on a multicopy plasmid, which should completely restore the wild-type phenotype of each individual mutation. In the case of an essential gene, with the wild-type gene supplied in trans during the “pop-in” phase of deletion construction, equal numbers of deletion and wild-type alleles should be recovered during the “pop-out” phase. However, such complementation experiments have been hampered by the dearth of selectable markers available historically in *Hbt. salinarum*. The widely used selection for novobiocin resistance is complicated by spontaneous resistance as well as detrimental growth effects in the minimal medium required for maintenance of the deletion plasmid.

We were able to delete all of the RPA homologs apart from *rfa2*, one of two eukaryotic-type large subunit homologs. This could potentially be due to a severe growth defect present in Δ*rfa2* cells, but that is unlikely given the high number of clones that were screened. However, we were able to successfully engineer an *rfa2* deletion in a strain with constitutively high *rfa3* operon expression, suggesting that high levels of one RPA may be able to compensate for the loss of another if the homology is sufficiently high. This was demonstrated in the related halophile *Hfx. volcanii*, although in that organism the *rfa1* homolog was found to be essential. Growth arrest following *rfa1* downregulation was partially suppressed by overexpressing the *rfa3* homolog despite the lack of homology between the two proteins [17]. It should be noted, however, that this was only discovered through deliberate overexpression, and such a suppressor was not isolated during construction of the deletion strains in *Hfx. volcanii*. In our suspected *rfa2* deletion strain, however, although *rfa2* expression was lower than in the wild-type, there was a detectable signal despite no amplification of the wild-type allele using primers outside of the transcript region. This suggests that the transcript is originating elsewhere in the genome through a possible rearrangement, underscoring its essentiality.

Our recovery of an otherwise wild-type strain carrying the *rfa1* deletion allele in *Hbt. salinarum* demonstrates its non-essential nature. Rfa1 appears to be important in more than just repair of damage, as the strain carrying this deletion demonstrated a severe growth defect. Despite the parallels in gene organization and high identity of the homologs, the roles that these proteins play in these two related organisms have diverged, underscoring the multiple roles that RPAs play in normal growth as well as in response to genomic stressors. Although there was no change in expression in this study under UV or mitomycin C treatment, this gene has increased expression after H_2_O_2_ treatment, suggesting a role in oxidative damage repair [33].

The expression of two bacterial-type SSB homologs (*rfa5* and *rfa6*) is particularly interesting given the identification of a bacterial-type SSB in human cells [9]. The human SSB homolog has been shown to be one of the first proteins to localize to sites of DNA double-strand breaks (DSB) and is an important player in the DNA damage response. In *Hbt. salinarum*, these homologs do not appear to play a major role in the repair of DSB induced by ionizing radiation, or in the repair of UV-induced damage, based on the lack of sensitivity to these agents in the deletion strains. However, they likely play some role in the repair of interstrand crosslinks induced by mitomycin C, as evidenced by sensitivity in the deletion strains and induction of expression. The conservation of these homologs (with 81% identity) on the minichromosomes, and across the Haloarchaea, would indicate their importance to the cell under certain conditions encountered on a regular basis.

Of note is the significant upregulation of *rfa2* expression, but not of the cotranscribed gene *rfa7*, following UV and mitomycin C treatment. This gene has also been demonstrated to be upregulated following gamma irradiation and H_2_O_2_ stress; in both cases, the accompanying genes also appear to not be regulated [33,34] The Rfa2 protein is suspected to be the primary RPA involved at the replication fork in *Hbt. salinarum* given the proposed essentiality of the gene. It may be surprising, then, that the expression levels rise so dramatically following DNA damage given the plethora of other proteins available to be involved in those repair pathways. However, in *Deinococcus radiodurans*, despite the presence of the IR-induced novel SSB DdrB, the replicative SSB is also induced following DNA damage [25,35]. Like SSB, Rfa2 may be a critical component of all replication and repair protein complexes.

The phosphoesterase component, *yhcR*, of the *rfa2* operon was assumed to be uninvolved in the repair pathways based on the IR and UV survival phenotype of the deletion strain. The drastic effect of deleting this gene on mitomycin C survival, but not from deletion of the co-transcribed *rfa7*, indicates an unanticipated relationship with repair of crosslinking damage. Phosphesterases from many genera of bacteria and archaea are part of a superfamily involved in non-homologous end-joining [36]. These results may indicate a similar function for the YhcR protein in *Hbt. salinarum*.

In most bacterial and eukaryotic organisms, a multifunctional RPA/SSB carries out the essential roles of DNA replication and repair. The euryarchaeota have evolved a unique set of proteins to carry out these functions [8] and this broad repertoire of RPA homologs may be a major determinant of the high tolerance of these organisms to genomic stress. The evolutionary conservation of the multiple homologs within the Haloarchaea indicates a possible adaptation unique to the saline environment. The difference in response of the various RPA deletions in *Hbt. salinarum* suggests a unique distribution of those functions and provides an opportunity to investigate the individual activities in separate proteins. A genetic analysis using strains containing multiple individual deletions would establish epistatic relationships as well as delineate the minimum repertoire of RPA genes necessary for carrying out life in *Hbt. salinarum*.

## Figures and Tables

**Table 1:** *Halobacterium salinarum* and *Haloferax volcanii* replication protein A (RPA)/single-stranded DNA binding (SSB) protein homologues, shared features, and percent identity.

	*Hbt. salinarum*		*Hfx. volcanii*				
RPA/SSB Type	Protein	Size (aa)	Protein	Size (aa)	Shared Features	% Identity	E Value
Eukaryotic	Rfa2	460	RpaA1	427	two OB-folds/ one Zinc Finger	64	1 × 10^−179^
	Rfa3	473	RpaB1	311	one OB-fold/ one Zinc Finger	67	2 × 10^−150^
	Rfa7	465	RpaA2	623	COG3390 domain; uncharacterized	65	8 × 10^−93^
	Rfa8	190	RpaB2	196	COG3390 domain; uncharacterized	75	2 × 10^−101^
	YhcR	247	HVO_1336	260	Phosphoesterase	57	1 × 10^−78^
	Ral	134	HVO_0290	137	Unique to Haloarchaea [18]	66	7 × 10^−65^
Crenarchaeal/Bacterial	Rfa5 Rfa6	330 299	HVO_A0019	302	1 OB fold	88 * 78	0.0 * 3 × 10^−173^
			HVO_A0374	301	1 OB fold	88 83	0.0 0.0
			HVO_A0409	287	1 OB fold	73 71	8 × 10^−155^ 4 × 10^−150^
Euryarchaeal	Rfa1	474	RpaC	483	3 OB folds; Essential in *Hfx. volcanii*	60	0.0

* First values are compared to Rfa5; second values are compared to Rfa6. OB: oligonucleotide oligosaccharide binding; aa: amino acids.

**Table 2:** *Hbt. salinarum* strains used.

Strain	Genotype	Source
NRC-1	Wild-type	Lab stock
LH5	Radiation-resistant mutant of NRC-1	[14]
LH101	As NRC-1 but Δ*ura3*	This study
LH102	As LH5 but Δ*ura3*	This study
LH110	As LH101 but Δ*rfa5*	This study
LH128	As LH101 but Δ*yhcR*	This study
LH134	As LH101 but Δ*rfa3*	This study
LH136	As LH101 but Δ*rfa8*	This study
LH138	As LH101 but Δ*ral*	This study
LH140	As LH101 but Δ*rfa1*	This study
LH142	As LH101 but Δ*rfa6*	This study
LH154	As LH102 but Δ*rfa2*	This study
LH166	As LH101 but Δ*rfa7*	This study

**Table 3:** Expression of *rfa2* in suspected *rfa2* deletion strain relative to the wild-type. Expression was normalized to *eef2* expression.

Strain	(*rfa2*)/(*eef2*)	Relative Expression
LH101	0.001	1.0
LH154	0.003	0.32

**Table 4:** Absolute mRNA levels of RPA genes.

Target	Quantity (Copies/µL)	Standard Deviation
*rfa1* (‡,¥)	3.22 × 10^7^	±1.48 × 10^7^
*rfa2* (‡,¥)	2.18 × 10^6^	±2.08 × 10^5^
*rfa3* (‡)	1.8 × 10^8^	±8.83 × 10^7^
*rfa5.6* (‡,¥)	4.88 × 10^6^	±1.07 × 10^6^
*rfa7* (‡,¥)	1.10 × 10^7^	±1.87 × 10^6^
*rfa8* (‡)	9.70 × 10^7^	±1.76 × 10^7^
*eef2*	2.69 × 10^8^	±5.9 × 10^7^

‡ *p* <0.05 compared to *eef2*, ¥ *p* < 0.05 compared to *rfa3.*

**Table 5:** Absolute levels and relative in expression of RPA genes 3 hours following treatment with UV-C or mitomycin C (MMC). Relative change to the untreated RPA genes based on expression values normalized to the corresponding maintenance gene *eef2* expression.

Gene	Treatment	Copies/µL	(Target)/(*eef2*)	Relative Change	*p*-Value
*rfa1*	Untreated	3.22 × 10^7^	0.12	1.0	
	UV-C	3.04 × 10^7^	0.10	0.81	0.76
	MMC	2.67 × 10^7^	0.08	0.65	0.90
*rfa2*	Untreated	2.18 × 10^6^	0.01	1.0	
	UV-C	1.97 × 10^7^	0.06	7.74	0.001
	MMC	1.93 × 10^7^	0.06	7.01	0.001
*rfa7*	Untreated	1.10 × 10^7^	0.04	1.0	
	UV-C	1.32 × 10^7^	0.04	1.03	0.65
	MMC	9.44 × 10^6^	0.03	0.68	0.92
*rfa3*	Untreated	1.79 × 10^8^	0.67	1.0	
	UV-C	3.57 × 10^8^	1.15	1.71	0.19
	MMC	2.54 × 10^8^	0.75	1.120	0.47
*rfa8*	Untreated	9.70 × 10^7^	0.36	1.0	
	UV	2.27 × 10^8^	0.73	2.01	0.33
	MMC	2.49 × 10^8^	0.73	2.03	0.03
*rfa5.6*	Untreated	4.88 × 10^6^	0.02	1.0	
	UV-C	9.92 × 10^6^	0.03	1.74	0.51
	MMC	1.70 × 10^7^	0.05	2.75	0.06

## Supplementary Materials

The following are available online at http://www.mdpi.com/2073-4425/9/4/223/s1, Figure S1: Verification of deletion of *rfa2* in LH154, Figure S2: Survival of additional *Hbt.salinarum* deletion strains to UV-C damage, Table S1: Primers used for deletion creation and verification, Table S2: Transcript primers used for transcript visualization of the *rfa2* operon. Table S3: qPCR primers and Taqman™ probes used in this study.
