# Supplementary material for: Divergent Roles of RPA Homologs of the Model Archaeon Halobacterium salinarum in Survival of DNA Damage
Source: Genes (Basel). 2018 Apr 20;9(4):223. doi: 10.3390/genes9040223 (PMC5924565; doi:10.3390/genes9040223)
Supplement: Supplementary file 1 [file genes-09-00223-s001.pdf]

genes
MDPI

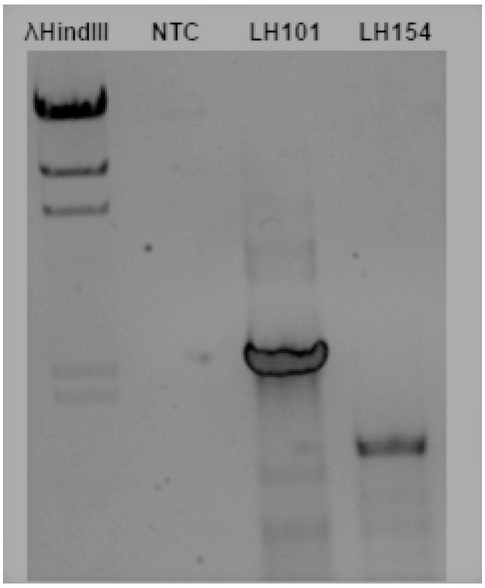

Figure S1. Verification of deletion of *rfa2* in LH154. Genomic DNA from LH101 (WT) and LH154 (LH102Δ*rfa2*) was used as template in PCR reactions with rfa2_UF and rfa2_DR primers. LH101 shows the expected 2581 bp wild-type band and LH154 shows the 1053 bp deletion band.

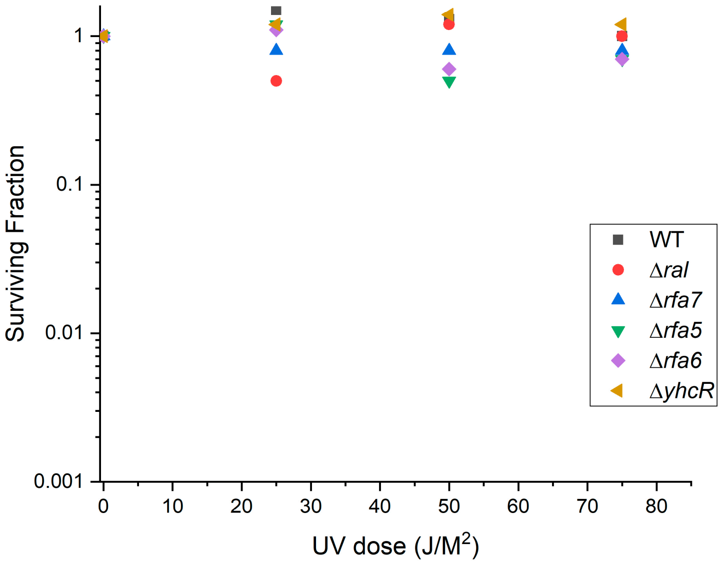

Figure S2. Survival of additional *Hbt. salinarum* deletion strains to UV-C damage. LH101 (WT), LH138 (Δ*ral*), LH166 (Δ*rfa7*), LH110 (Δ*rfa5*), LH128 (Δ*yhcR*) and LH1142 (Δ*rfa6*).

Table S1. Primers used for deletion creation and verification.

Primer	Sequence	Source
rfa1_UF	GCGCGAATTC[1] ACCGCCTGTGGGTCGTGATCG	This study
rfa1_UR	CCTCGACGCGAGTCACGACTCGCCCATTGTCGGTTCAGGCC	This study
rfa1_DF	GGCCTGAACCGACAATGGGCGAGTCGTGACTCGCGTCGAGG	This study
rfa1_DR	CGCGGAATTCGTATGTCTTCGGCTTGGACGGTGC	This study
rfa3_UF	GCGCGAATTCGGCAGACAGAGCATGACGGCG	This study
rfa3_UR	CGCCGCTCATTTCAGATCGACCGGTCGCTCATGGTCGTGTATCCG	This study
rfa3_DF	CGGATACACGACCATGAGCGACCGGTCGATCTGAAATGAGCGGCG	This study
rfa3_DR	CGCGGAATTCGCTCGGCGTACTCGTTGCCC	This study
rfa8_UF	GCGCGAATTCCAGGGCCGATACTCGGTGAAGC	This study
rfa8_UR	GCGTTTGTCGCCCTCACTCCGCGCTCATTTCAGATCGACCTCGC	This study
rfa8_DF	GCGAGGTCGATCTGAAATGAGCGCGGAGTGAGGGCGACAAACGC	This study
rfa8_DR	CGCGGAATTCCGCCAGCCGGAAGAAACGCG	This study
ral_UF	GCGCGAATTCCGCTGTACGCGTTGCTCCCG	This study
ral_UR	CCGCCTATCCGAGCTGGTAGGTCCCCATGTACCGGACAGTCGC	This study
ral_DF	GCGACTGTCCGGTACATGGGGACCTACCAGCTCGGATAGGCGG	This study
ral_DR	CGCGGAATTCTACGCTTTTCCACTAACCCGCCG	This study
rfa5_UF	GCGCGAATTCGAGACATCGTCTCCCGGTAACCG	This study
rfa5_UR	GGTTGCGTCAGTTGGCTATGCTTCCCCCGACAGAACCATCTGCGCGCTCT	This study
rfa5_DF	AGAGCGCGCAGATGGTTCTGTCGGGGGAAGCATAGCCAACTGACGCAACC	This study
rfa5_DR	CGCGGAATTCCACAGTACGCACAAAGTGCCTGC	This study
rfa6_UF	GCGCGAATTCTTCGCGTCGCTCGCGACCG	This study
rfa6_UR	GCGCCGACTGGCTATTCGTCCCTTGACATTGGAACTTAGACCAGTTCC	This study
rfa6_DF	GGAACTGGTCTAAGTTCCAATGTCAAGGGACGAATAGCCAGTCGGCGC	This study
rfa6_DR	CGCGGAATTCTCGTAGCGCCGGATCTCGGC	This study
*rfa2*_UF	GCGCGAATTCGCGACGAACACGGACAGCTCG	This study
*rfa2*_UR	TCGCTCATGCGTCCACCTCCGCCGCGACTGTATGCATGTTGTCG	This study
*rfa2*_DF	CGACAACATGCATACAGTCGCGGCGGAGGTGGACGCATGAGCGA	This study
*rfa2*_DR	CGCGGAATTCTCGACGCGGTCGTGGGCCG	This study
*rfa7*_UF	GCGCGAATTCACGAGGTCGAGGTCGGCGGG	This study
*rfa7*_UR	AGCGCCATCAGATGGCTTTCAGGCCGTCGCTCATGCGTCCACCTC	This study
*rfa7*_DF	GAGGTGGACGCATGAGCGACGGCCTGAAAGCCATCTGATGGCGCT	This study
*rfa7*_DR	CGCGGAATTCTCGTCGGTCAACCGCACCGC	This study
*yhcR*_UF	GCGCGAATTCATCCGAGACGGCGTCGCCG	This study
*yhcR*_UR	CGGTCGCGTCGGCTACACCCCGGGCTCGACGAGCGCCATCA	This study
*yhcR*_DF	TGATGGCGCTCGTCGAGCCCGGGGTGTAGCCGACGCGACCG	This study
*yhcR*_DR	CGCGGAATTCTCGACGTCTGCAAGTCCATCCC	This study

[1] UF and DR primers have an EcoRI restriction site (underlined) engineered onto the 5′ end for ligation into their corresponding plasmid.

Table S2. Transcript primers used for transcript visualization of the *rfa2* operon.

Primer	Sequence	Purpose
LCDrfa27upF	GTGGTCGCCGACACCATC	Amplification of the *rfa2*/*rfa7* junction
JPrfa72dnR	CACCACGTCGTCGTTGAC	
JPrfa7rupF	CGCAGTGAGGTGGTGTC	Amplification of the *rfa7*/*yhcR* junction
LCDyhcR7dnR	GTGTGTCCGTGTGCGAAC	

Table S3. qPCR primers and Taqman™ probes used in this study.

Primer Name	Sequence	Source
*rfa1*_right	CCCACCACGAGATTCGACAC	This study
*rfa1*_left	GACCTCTCGTTGGGGGTTTC	This study
*rfa1*_probe	CCTGGACACCGAGGCCGTCCGG	This study
*rfa2*_right	CCACGAGGTTCTGCAGGTTC	This study
*rfa2*_left	CAACCCTTATGCCCCAGGAC	This study
*rfa2*_probe	CGCCGAGGAGCTCGCCTCCGAC	This study
*rfa3*_right	GACTTCGACGGTGTCGTTGC	This study
*rfa3*_left	GGAAGCACGGCTGGAGAAAC	This study

*rfa3*_probe	CCCGGTCGACGAGGCCCGCC	This study
*rfa7*_right	AAGACGCCCACGACGAACA	This study
*rfa7*_left	GACGACGCCGACTTCTCGTA	This study
*rfa7*_probe	CGCCGAACTACGTGGTGACGCCG	This study
*rfa8*_right	TCGTTGCCCTCCTCGTTGAAG	This study
*rfa8*_left	CGATCACGATGGTCGAGGAG	This study
*rfa8*_probe	CGAGGCCGCCGAGCGCACGC	This study
*rfa56*_right*	CCCGTTCGGGGTCGACCAT	This study
*rfa56*_left*	GGAGCGTATTCGGGCGCAGGAA	This study
*rfa56*_probe*	GGCCGAGCTGGGAACGCAGGACGG	This study
*eef2*_right	TGTCAGTGAGGGTGGTTTTTCC	This study
*eef2*_left	ACGAAAGAAGATTGTCGAACAGTG	This study
*eef2*_probe	AACGGCTGATGGACAACCCGGAGC	This study
